# Biomarkers in Neurodegenerative Diseases: Proteomics Spotlight on ALS and Parkinson’s Disease

**DOI:** 10.3390/ijms23169299

**Published:** 2022-08-18

**Authors:** Rekha Raghunathan, Kathleen Turajane, Li Chin Wong

**Affiliations:** Bioanalytical and Biomarker, Prevail Therapeutics, a Wholly Owned Subsidiary of Eli Lilly and Company, New York, NY 10016, USA

**Keywords:** biomarkers, neurodegeneration, ALS, PD, TDP43, proteomics, LC-MS/MS

## Abstract

Neurodegenerative diseases such as amyotrophic lateral sclerosis (ALS) and Parkinson’s disease (PD) are both characterized by pathogenic protein aggregates that correlate with the progressive degeneration of neurons and the loss of behavioral functions. Both diseases lack biomarkers for diagnosis and treatment efficacy. Proteomics is an unbiased quantitative tool capable of the high throughput quantitation of thousands of proteins from minimal sample volumes. We review recent proteomic studies in human tissues, plasma, cerebrospinal fluid (CSF), and exosomes in ALS and PD that identify proteins with potential utility as biomarkers. Further, we review disease-related post-translational modifications in key proteins TDP43 in ALS and α-synuclein in PD studies, which may serve as biomarkers. We compare relative and absolute quantitative proteomic approaches in key biomarker studies in ALS and PD and discuss recent technological advancements which may identify suitable biomarkers for the early-diagnosis treatment efficacy of these diseases.

## 1. Introduction

Amyotrophic lateral sclerosis (ALS) and Parkinson’s disease (PD) are neurodegenerative diseases characterized by the progressive degeneration of neurons. 

In ALS, the progressive degeneration of motor neurons occurs with a debilitating loss of movement control. The majority (90–95%) of ALS cases are sporadic in origin and 5–10% of cases are of known genetic origin [1]. Of the known genetic cases, mutations in the *C9ORF72*, *SOD1*, *TARDBP* [2,3,4], *NEK1* [5,6], *UBQLN2* [7,8], *KIF5A* [9,10], and *FUS* [11,12] genes contribute to familial ALS cases [13]. Other genes that contribute to familial ALS includes *VCP, ALS2, SETX*, *ANG*, *PFN1*, *MATR3*, *CHCHD10*, *TUBA4A*, *TBK1*, *GRN*, *C21orf2*, and *OPTN* [14].

In PD, the progressive degeneration of dopaminergic neurons occurs with a loss of movement control. The majority (90%) of PD cases are sporadic in origin and 10% of cases are of known genetic origin [15]. Risk for familial PD has been associated with 28 distinct chromosomal regions called *PARK* genes [16,17,18,19]. Six genes are linked with monogenic familial PD. They include *SNCA* and *LRRK2* mutations that have been linked with an autosomal dominant form and *Parkin*, *PINK1*, *DJ-1*, and *ATP13A2*, which have been linked with an autosomal recessive mode of inheritance in familial PD. Variants in certain other genes such as *UCHL1*, *GAK*, *MAPT*, *GBA*, *NAT2*, *INOS2A*, *GAK*, *HLA-DRA*, and *APOE* are associated with increased risk of PD, where additional factors, along with mutations, might play a role in disease causation [20].

Biomarkers are essential for the early diagnosis, prognosis, and assessment of treatment for both ALS and PD, but biomarker discovery has been challenging for these diseases.

The increased use of unbiased tools including proteomics, however, is advancing biomarker discovery. Proteomics enables biomarker discovery using traditional mass spectrometry-based platforms as well as newer platforms such as multiplexed immunoassays and aptamer techniques by identifying disease-associated protein alterations. Here, we provide a detailed review of several key proteomics studies showing promising techniques and describe the technological advancements for ALS and PD biomarker discovery.

## 2. Overview of Proteomics Technological Advancement for Biomarker Discovery

**Figure 1:**
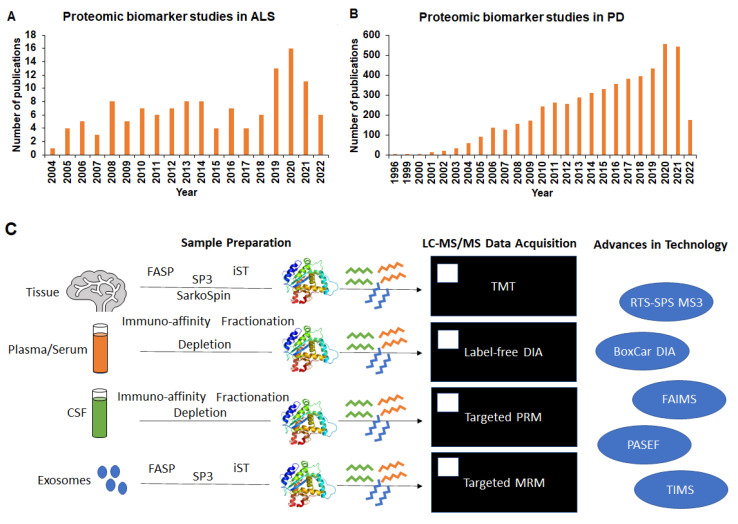
Summary of the number of proteomic biomarker studies in (**A**) ALS and (**B**) PD. (**C**) Schematic of proteomics workflow and advances in mass spectrometry instrumentation for biomarker discovery.

Proteomics tools enable the deep protein profiling of tissue, plasma, serum, and CSF for biomarker discovery [21]. Using proteomics, several studies have attempted to identify pathological disease mechanisms by identifying differentially expressed proteins and proteoforms (proteins and their modifications) as potential biomarkers in disease models, biofluids from patients, as well as post-mortem brain tissue. Discovery proteomics technology using mass spectrometry, immunoassays, and aptamers can identify several thousands of differentially expressed proteins and their altered forms with disease. However, only a fraction of them have translated to clinical utility due to the lack of standardized validated assays and the lack of access to instrumentation. Several studies that assessed specific brain regions for biomarker discovery fail to recognize the systemic mechanisms in neurodegenerative disease progression. Many neurodegenerative disease mechanisms converge and present differently. However, some have similar affected underlying pathways which makes the identification of disease-specific biomarkers a challenge. The gap needs to be bridged between the increasing number of publications studying biomarkers for ALS and PD (Figure 1A,B) and relatively few biomarkers in the clinic.

Some of the technological advances in mass-spectrometry-based proteomics for biomarker discovery are described in Figure 1C and Table 1. 

Improved instrumentation, including faster scan speeds, parallel accumulation serial fragmentation (PASEF) [22,23], and trapped ion mobility [24,25], enables the identification of disease-associated proteins and their modifications from patient samples using small sample volumes. While these technological advances drive the limits of quantitation and enable sensitive analysis to gain the greatest depth in proteome being measured, the need to analyze thousands of samples robustly still remains a challenge in biomarker discovery.

Improvements in data acquisition strategies include using label-free data-independent acquisition (DIA), making the relative quantitation of proteins possible from many samples. In the DIA approach, complete data are acquired using a label-free strategy matched to a large spectral library built from representative samples of the cohort. While previously DIA required building and using a spectral library for the accurate identification of the acquired MS2 spectra [26], recent advances in direct DIA and machine learning algorithms allow library-free accurate identification [27] and quantification [28]. Today, >10,000 proteins from tissue [29] or >1000 proteins from plasma can be identified by DIA [30]. To overcome the dynamic range challenge and gain depth in the number of proteins identified from complex matrices such as plasma/serum, using sample preparation strategies including depletion, immuno-enrichment, and data acquisition strategies (such as the BoxCar-segmented MS1 approach and BoxCarmax) can achieve maximized depth in the proteome [31,32,33].

An alternative relative quantitative strategy uses isobaric TMT tags. Complete deep data profiles for proteins are acquired in the MS2 dimension and enhanced quantitation using synchronous precursor selection (SPS) in the MS3 dimension overcomes inherent challenges with TMT such as ratio compression. Currently, the use of real-time search SPS MS3 gives the accurate quantitation and in-depth profiling of pooled fractionated samples without any sacrifice in the number of proteins identified, overcoming previous limitations in TMT-based approaches. Using automated sample preparation, ion mobility with high-field asymmetric waveform ion mobility spectrometry (FAIMS), and real-time search SPS MS3, deep protein profiles from challenging matrices such as plasma can be obtained [34,35].

Further, absolute quantitation strategies using targeted proteomics approaches to monitor selected peptides or proteins by parallel reaction monitoring (PRM) or multiple reaction monitoring (MRM) across large study cohorts identified biomarkers are validated for clinical use [36]. An advancement in this field includes the improved sensitivity in targeted analysis platforms.

Traditional nano-LC approaches allow a small number of samples to be analyzed per day, making rapid and robust analysis of large cohorts for biomarker discovery challenging. An advancement in throughput involves the use of novel LC platforms such as Evosep which can analyze up to 300 samples per day in a rapid and ultra-robust analysis compared to traditional nano-LC approaches [37]. Using this platform makes clinical proteomic analysis for biomarker discovery possible on a large scale. 

Other non-mass-spectrometry-based proteomics techniques for biomarker discovery include O-link and aptamer-based technology. O-link technology uses a multiplexed immunoassay and has the advantage of overcoming the dynamic range challenge via the enrichment of proteins with antibodies, but is limited in the panel of proteins that can be detected because it relies on the availability of specific antibodies [38]. Aptamer technology involves small nucleic acids binding to proteins and subsequent detection by means of complementary nucleic acids and fluorescence [39,40]. While these approaches have high throughput, they rely on the availability of well-characterized reagents, unlike mass spectrometry which is an unbiased proteomic approach. Combining these complementary approaches of mass-spectrometry-based proteomics, immunoassays, and aptamers can help to gain depth in the proteins identified [41]. 

## 3. Proteomics for Biomarker Discovery in ALS 

### 3.1. Differential Expression of Proteins and Interactome from Post-Mortem Human Tissue as Biomarkers in ALS 

Post-mortem tissue proteomics is a key method used for identifying disease biomarkers in bulk tissues, tissue sections on a slide, and single cells. Several workflows have been suggested for bulk tissue analysis such as suspension trapping filter-aided sample preparation (FASP), single-pot solid-phase-enhanced sample preparation (SP3), and in-StageTip (iST) [42] for disease biomarker analysis. On a FFPE/frozen slide, proteins associated with pathological regions in the tissue can be identified by liquid chromatography–tandem mass spectrometry (LC-MS/MS) or matrix-assisted laser desorption–ionization (MALDI) approaches [43,44,45,46]. Using a combination of imaging and high-resolution mass spectrometry, the characterization of proteins at a single cell resolution and the intracellular sub-proteome has been possible [47,48]. This has been utilized to study the sub-proteome of endosomal vesicle trafficking [49]. Since these endo-lysosomal networks are known to be dysregulated in neurodegenerative diseases, the signatures of potential biomarkers can be detected [50]. These approaches enable biomarker discovery from tissue.

Studies that provide insight into potential tissue markers in ALS are summarized in Table 2. In ALS, proteomic profiling via LC-MS/MS in post-mortem spinal cord tissue compared sporadic ALS and controls, revealed ATP5D, and calmodulin was downregulated in sporadic ALS [51]. Here, 2D-gel electrophoresis combined with LC-MS/MS was used in identification of biomarkers. While 2D gel electrophoresis enables the isoelectric separation of proteins, the method is relatively low throughput and requires expertise to isolate spots consistently. By assessing ALS mutation carriers (C9orf72, SOD1, and TARDBP), sporadic ALS and controls identified several proteins that were upregulated, including UCHL1, MAP2, CAPG, GPNMB, HIST1H4A, HIST1H2B, NEFL, NEFH, NEFM, CHIT1, and CHI3L1 in both spinal cord and CSF in ALS [52]. The upregulation of four of these proteins (UCHL1, MAP2, CAPG, and GPNMB) in both CSF and spinal cord was confirmed in an independent cohort by MRM [52]. CSF from symptomatic and asymptomatic ALS mutation carriers was assessed, and NEFL, NEFM, NEFH, CHIT1, and CHI3L1 were upregulated in symptomatic individuals [52]. The strength of this analysis lies in the use of both post-mortem spinal cord and CSF in discovery proteomics. The CSF proteomics method was modified to enhance depth. Further, candidate markers were validated by absolute quantitation using a targeted proteomic approach in an independent cohort. Using both discovery and targeted proteomics approaches to validate markers in an independent cohort, this study provides a comprehensive and validated approach in identifying biomarkers. Proteomics is also valuable in assessing the interactome. A proximity-based ligation BioID assay of di-peptide repeats in ALS patients with *C9ORF72* mutations identified chaperone proteins to be associated with poly-GA, while ribosomal and nucleolar proteins were associated with poly-GR and poly-GP di-peptide repeats in ALS patients [53,54]. Toxic repeats can be identified using BioID to analyze proteins which provide insight into disease mechanisms. A disadvantage of the method is the need to express a vector and identify interacting proteins. This approach is possible in model systems and primary cells, but not in patient samples. In these references, primary proteins interacting with poly-GA, poly-GR, and poly-GP repeats were identified in cortical neurons and cells, and these were validated in patient samples by mass spectrometry. This is a balanced approach in addressing disease mechanisms in model systems and confirms the findings in patient samples. 

### 3.2. Post-Translational Modifications in TDP43 from Tissue Proteomics Studies in ALS

Post-translational modifications (PTMs) include changes in the amino acid side chains in proteins due to the covalent addition of functional groups or proteolytic cleavage [64]. PTMs are readily detected by mass spectrometry due to characteristic mass shifts caused by the biochemical modification of proteins [65]. Abnormal PTMs can disrupt normal biological processes and serve as biomarkers for disease diagnosis and progression [66,67].

**Figure 2:**
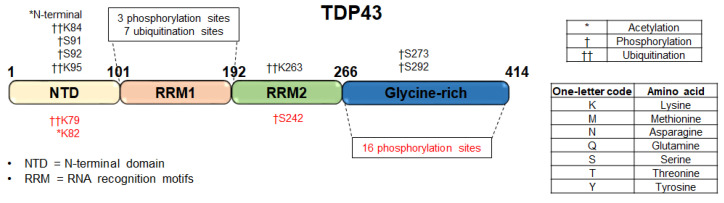
Schematic representation of post-translational modifications (PTMs) in TDP43 in ALS. The red font indicates putative disease-specific modifications [70] and the black font indicates all known modifications from proteomics databases (ProteomicsDB and PhosPhosite) and literature.

A pathological hallmark of ALS is the presence of cytoplasmic inclusions of TAR DNA-binding protein 43 (TDP43) and mislocalization of the protein from the nucleus to the cytoplasm [68]. Major modifications in TDP43 associated with protein aggregates include truncations, ubiquitination, and hyperphosphorlation in the C-terminal and N-terminal domains [69,70] (Figure 2). 

PTMs in pathological TDP43 were differentiated from endogenous TDP43 using a method called SarkoSpin (a technique for the isolation of pathological TDP43 aggregates), and characterized by mass spectrometry from ALS brains [71]. Using SarkoSpin on ALS and FTD brains, normal proteins including physiological TDP43 were exclusively found in the supernatants, while protein aggregates such as pathological TDP43 that underwent polyubiquitation and hyperphosphorylation were detected in the pellets. SarkoSpin is an excellent method used to separate insoluble disease-specific aggregates from the soluble form and could potentially be applied to other aggregated proteins common in neurodegenrative diseases. Here, after separating the pathological TDP43 aggregates, LC-MS/MS with spectral counting was used for the relative quantitation of proteins in different groups. A label-free approach allows intact PTMs to be evaluated, in turn idenitfying and quantifying the relative abundance of proteins in different disease groups. Phosphorylated TDP43 leads to an increase in cytoplasmic and mitochondrial mislocalization and aggregation in neurons. The ubiquitination of TDP43 has been associated with TDP43 aggregation [72]. These modifications that are unique to disease pathology could serve as biomarkers for ALS. Most of the modifications and cleavages occurred in the glycine-rich C-terminal and N-terminal domains of TDP43 [70]. In ALS, a measured ratio of C:N terminal TDP43 fragments >1.5 could differentiate ALS from control subjects in a study using post-mortem brain and spinal cord tissue [55]. 

### 3.3. Plasma and Serum as Sources for Proteomic Biomarkers in ALS

There are several strategies for plasma protein profiling in disease biomarker identification. The first technique presented here uses mass-spectrometry-based proteomics in plasma. To successfully identify disease biomarkers from plasma, it is important to find ways to measure the low-abundance disease-relevant proteins, while overcoming the large dynamic range of proteins in plasma. This is challenging, since most instruments can measure a range of up to 5 orders of magnitude, with bias towards the most abundant proteins, while proteins in plasma span 10 orders in magnitude, with disease-specific proteins being lower in abundance. The depletion of the most abundant proteins [73,74], the enrichment of proteins of interest with antibodies [75], and deep offline fractionation are commonly used methods [75] to identify low-abundance disease-relevant proteins. While these methods increase depth, they result in lower throughput [76]. These strategies have identified clusterin and ficolin-3 as differentially expressed proteins in ALS plasma [57,58]. Here, SWATH was applied for the identification of proteins, and levels were confirmed by Western blotting. SWATH has the advantage of being label-free, and can preserve modifications in proteins for later query if needed and provide relative quantitation. The disadvantage of SWATH is the requirement of careful design in windows to gain the maximum number of proteins identified, thus involving the need for complex software to interpret the spectra. The advantages of being unbiased and label-free, as well as providing complete data with minimal missing values, outweigh these disadvantages. The deep plasma profiling of matched plasma and CSF samples showed that the upregulation of gelsolin and several proteins such as chitinase-3-like 1 and alpha-1 antichymotrypsin was validated in ALS plasma and CSF [56]. Here, matched plasma and CSF samples were analyzed by discovery proteomics. The advantage of using matched samples enables the identification of neurological and systemic changes in proteins due to the disease. Label-free quantitation provided a relative abundance of differentially expressed proteins. Using machine learning algorithms, two candidate proteins were validated for absolute levels by a targeted proteomics approach. This strategy uses both discovery and targeted proteomics with machine learning to select candidate markers which provides a well-defined strategy for biomarker discovery.

Another strategy to gain depth without necessarily compromising throughput involves using the TMT calibrator method, where spiking the disease peptides from peripheral blood mononuclear cells (PBMCs) or brain tissue in multiplex channels of TMT boosts the associated plasma proteome being measured in the other channels. ALS studies have used this technique to study the rate of disease progression [57,58] and phenotypic variability in sporadic ALS patients [77,78], and have identified proteins in biological processes of senescence, RNA processing, cell stress, and metabolism; moreover, major histocompatibility complex-II-linked immune reactivity and apoptosis were enriched in fast-progressing ALS. While using a booster channel from tissue or PBMC is advantageous in boosting low-abundance peptide signals seen in biofluids that are otherwise masked, the validation of positive identification is required. Quantitation using isobaric tags enables multiplexing and depth gains. However, this method suffers from ratio compression which leads to inaccurate quantitation. The development of novel strategies such as real-time search MS3 overcomes some of the previous limitations in TMT-based quantitation. The known plasma biomarkers in ALS from discovery proteomics approaches are summarized in Table 2.

### 3.4. Cerebrospinal Fluid (CSF) Proteomic Biomarker Identification

CSF analysis is widely used in biomarker studies of neurodegenerative disease, since CSF is believed to reflect brain processes within the blood–brain barrier. Important considerations in CSF proteomics include the proper collection and storage of samples to minimize blood contamination and maximize protein stability [79]. In CSF, as with plasma, depletion [80], immuno-enrichment, and fractionation have been applied in several studies to acquire deep CSF profiles in ALS. 

Using these approaches, differentially expressed proteins in CSF from ALS patients have been identified as potential biomarkers (Table 2). Using isobaric tags for relative quantitation, nine proteins were upregulated in CSF in C9orf72 variant-associated ALS compared to controls. These include chitinase-3-like protein 2 (CHI3L2), alpha-crystallin B chain (CRYAB), profilin-1 (PFN1), transferrin receptor protein 1 (TFRC), triggering receptor expressed on myeloid cells 2 (TREM2), thioredoxin domain-containing protein 17 (TXNDC17), ubiquitin carboxyl-terminal hydrolase isozyme L1 (UCHL1), CHIT1, and NEFM [60]. Here, isobaric tags were used for the relative quantitation of differentially expressed proteins and eight candidate proteins were validated using a targeted proteomics approach for absolute levels. Multiplexing can be used to assess large study samples, but requires a good study design to bridge the different TMT batches. Following up the discovery proteomics with validation in an independent cohort can confidently and effectively identify biomarkers. Other studies have also demonstrated the upregulation of chitotriosidase, chitinase-3 like protein 1, chitotriosidase-3 like protein 2, chitotriosidase-1 (CHIT1), alpha-1-antichymotrypsin, and amyloid beta A4 protein [56,59,81] in ALS. In these studies, a label-free strategy was applied for the discovery of biomarkers, and a small number of candidates were validated in a separate cohort using targeted proteomics or ELISA. Label-free quantitation allows post-translational modifications to be preserved and is a great method to overcome study design biases that may occur in TMT-based workflows. Validating proteins observed in discovery proteomics using a targeted or orthogonal approach provides greater confidence in the identified biomarkers. 

### 3.5. Exosomes Proteomics in Biomarker Identification

Exosomes are extracellular vesicles released from various types of cells, including CNS cells, and are enriched in a variety of bioactive molecules such as RNAs, proteins, and lipids [82]. Exosomes carrying cell-type-specific molecules reach the periphery by crossing the blood–brain barrier (BBB), making them ideal for biomarker studies [82]. Measuring biomarkers in neuron-derived exosomes in plasma could serve to monitor neuronal health and neuroinflammation [83,84]. Indeed, profiling CSF exosomes from sporadic ALS patients identified three downregulated proteins and eleven upregulated proteins [61]. A label-free proteomics study in CSF exosomes demonstrated the upregulation of ubiquitin-like modifying-activating protein 1 (UBA1) in C9orf72-ALS patients [61]. Exosomes have been studied in neuroinflammation and disease progression biomarkers using proteomics in ALS. Here, a label-free strategy was applied for differential proteomic analysis. This strategy has the advantage of greater sensitivity in small samples, such as exosomes, and can preserve modifications seen in proteins. Here, only relative quantitation with proteomics was applied. If these results were validated in an independent cohort using a targeted or orthogonal approach, greater confidence in the markers could be established.

## 4. Proteomics for Biomarker Discovery in PD 

### 4.1. Differential Expression of Proteins from Post-Mortem Human Tissue as Biomarkers in PD 

Several studies investigated differentially expressed proteins that change post-mortem tissue with PD. In a proteomic study profiling the post-mortem substantia nigra of PD patients, pathway analysis found alterations in proteins associated with mitochondrial dysfunction, oxidative stress, or cytoskeleton impairment [85]. Here, the samples were analyzed using isobaric tags and some markers were validated by immunohistochemistry and Western blot. To gain depth, offline isoelectric focusing was used. While it is great that the markers were validated using an orthogonal method, all the quantitation was carried out using relative quantitative approaches. The implementation of an absolute quantitation method might be helpful to gain accuracy in the quantified biomarkers. In another proteomic study used to identify pathways that contribute to Lewy body (protein inclusions containing aggregated proteins) pathology, pathways such as Arp2/3, synaptic function, and hydrogen peroxide metabolism were found to be directly correlated with Lewy body pathology, while poly(A) RNA binding protein pathways such as TDP43 and FUS were inversely correlated with Lewy body pathology. In a comparison of Lewy body pathology with and without neuronal loss, it was found that CD59 was upregulated and RGS6 and GANAB were downregulated [86] (Table 3). Here, label-free quantitation was performed to assess pathways that contribute to Lewy body pathology. Further, pathways were identified using the quantitative proteomics results and rigorous statistics to define pathways and proteins associated with Lewy body pathology.

### 4.2. Post-Translational Modifications in Key Proteins from Tissue Proteomics Studies in PD

**Figure 3:**
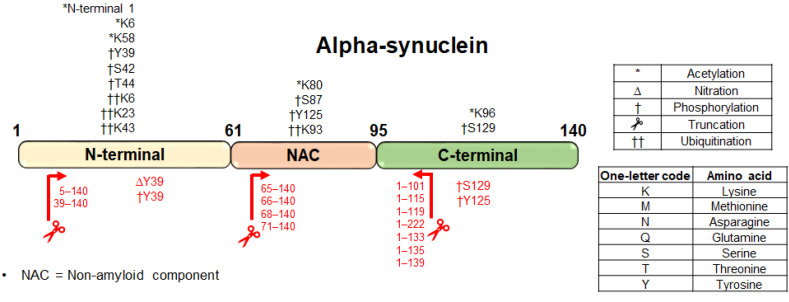
Schematic representation of post-translational modifications (PTMs) identified in α-synuclein. The red text indicates putative disease-specific modifications reported in PD [98,99,100,101,102,103,104,105,106,107,108,109,110], while black test indicates all known modifications that are reported in the literature and proteomic databases (ProteomicsDB and PhosPhosite).

Aggregated forms of α-synuclein found in Lewy bodies are a primary hallmark of PD. α-Synuclein has been associated with many PTMs, including acetylation, phosphorylation, nitration, O-GlcNAcylation, SUMOylation, and truncations. These modifications are linked to the aggregation and toxicity of α-synuclein [97] (Figure 3). 

Mass-spectrometry-based proteomic studies in human brains with synucleopathies reveal that the phosphorylation of α-synuclein at Serine 129 (Ser129-p) is the predominant modification method of α-synuclein in Lewy bodies, followed by ubiquitination and truncations at the C-terminus [98,101,110]. Here, by using a combination of detergents to extract the insoluble aggregates, Ser129-p was identified with MALDI. MALDI undergoes a relatively gentle ionization method, enabling the preservation of the phopho-group, and is more robust to the presence of detergents in the samples. The disadvantage of MALDI is that it can only identify few of the most abundant peptides or proteins. A study characterizing the interplay between tyrosine phosphorylation and nitration in the C-terminus found that phosphorylation at the proximal tyrosine 125 (pY125) altered metal binding and induced pathogenic aggregation [107]. Protein tyrosine nitration (PTN) at Y39 is also believed to be critical in oligomer formation [98,99,100,101,102,103,104,105,106,107,108,109,110]. 

PD, the process by which oligomeric proteins are seeded and spread, involves intricate interactions with the cell membrane and extra-cellular matrix (ECM), and these processes can be characterized in tissue proteomic studies. Glycosylation is the predominant post-translational modification in matrisome molecules (proteins constituting the cell membrane and ECM). Proteomics methods to study the glycosylation of matrisome molecules have been summarized by Raghunathan et al. [111]. A study in human prefrontal cortex identified that ECM molecules exhibit the highest degree of upregulation in PD. These molecules include proteoglycans associated with perineuronal nets and various collagen types [112]. They contribute to the blood–brain barrier and have important neuronal signaling implications. In addition, collagen type I has a differential hydroxyl proline state in PD compared to controls [113]. 

### 4.3. Plasma and Serum as Sources for Proteomic Biomarkers in PD

In PD, plasma levels of α-synuclein pS129 in α-synuclein are associated with motor symptom severity and disease progression [90,91]. Here, targeted proteomics is used to gain the absolute quantitation of pS129 in α-synuclein in plasma. One advantage is that it selectively monitors target peptides, overcoming other dynamic range challenges and providing absolute quantitation. However, it can be used on a limited number of targets. 

A metabolomic and proteomic study in plasma using mass spectrometry revealed that all apolipoprotein isoforms were downregulated in PD [89]. Assessing both metabolomic and proteomic analysis in plasma provides valuable information about the disease. Label-free proteomics analysis was used, and all quantitation was carried out with relative abundance. The validation of these markers in an independent cohort could strengthen these findings. In a systematic blood-based biomarker review in PD, seven proteins (apolipoprotein A1, apolipoprotein-A IV, inter-alpha-trypsin inhibitor heavy, complement C4A, complement C4B, complement C3, and haptoglobulin) were consistently downregulated in PD compared to the control [87]. Five proteins (clusterin, transthyretin, zinc α-2-glycoprotein, vitamin D binding protein, and afamin) were consistently upregulated in plasma and serum in PD compared to controls [87].

Aptamer-based approaches can overcome the dynamic range challenge associated with protein measurements in plasma by specifically enriching proteins that bind aptamers. Using aptamer technology, a multicohort study of plasma blood-based biomarkers was analyzed in 96 PD patients and 45 neurological controls. Four proteins (bone sialoprotein, osteomodulin, aminoacylase-1, and growth hormone receptor) were differentially expressed in ALS [114]. Aptamer-based technology overcomes the dynamic range challenge associated with plasma proteomics. Yet, it is limited by the panel of observable proteins that are predetermined, as well as by the specificity of the aptamers. The use of well-characterized reagents is necessary to rule out false positives with the aptamers. 

### 4.4. Cerebrospinal Fluid (CSF) as a Source for Proteomic Biomarkers in PD

Targeted proteomic identification and quantification in CSF was used as a diagnostic marker and marker of disease progression [90]. Using a targeted proteomics approach to monitor α-synuclein peptide (81–96) revealed that monitoring this peptide level in CSF can act as both a diagnostic marker and a marker of disease progression [90]. In a separate study, it was found that the level of pS129 in α-synuclein correlated with disease severity in PD [91]. Using a targeted proteomics approach enables absolute quantitation, and thus longitudinal comparisons in different cohorts. A global proteomics study using label-free quantitation in CSF identified multiple proteins downregulated in PD, including seven of eight members of the granulin family [92] (Table 3). 

### 4.5. Exosomes Proteomics in Biomarker Identification

In PD, exosomes have been investigated as a source of biomarkers (Table 2). Neuronal exosomes isolated from serum were investigated for signatures in clinical PD, finding that α-synuclein and clusterin levels together can serve as a marker for the differential diagnosis of PD [93]. While total plasma levels of α-synuclein show no change, neuronal exosomal α-synuclein correlate with disease progression in PD, indicating that an assessment of neuronal exosomes from plasma may be a superior strategy for biomarker discovery in other neurodegenerative diseases [115]. In addition, oligomeric α-synuclein resistant to proteinase K and pSer129 in α-synuclein was identified in plasma exosomes from PD patients by proteomics. The ratio of α-synuclein oligomer–total α-synuclein and the ratio of p-α-synuclein oligomer–total p-α-synuclein in plasma exosomes served as a diagnostic biomarker in PD compared to controls in a proteomic study [116]. In a PD study, α-synuclein levels were identified in L1CAM+ exosomes and correlated with GCase activity in PBMC [96]. Here, L1CAM+ exosomes were used as a marker of neuronal exosomes. There is still uncertainty around the specificity of L1CAM as a marker of neuronal origin. The measurement of oligomeric α-synuclein in plasma exosomes is novel and can be insightful if validated as a biomarker. Total plasma exosomes from PD patients in stage II and III were profiled by proteomics, and three proteins (clusterin, complement C1r, and apolipoprotein A1) were found to be downregulated in PD compared to control [94]. Here, by using a combination of 2D gel electrophoresis and MALDI-TOF, the relative quantitation of proteins was analyzed. MALDI provides few identified proteins and a greater depth, but lower throughput could be achieved using LC-MS/MS. Further, these biomarkers only have relative quantitation. A follow-up study with targeted proteomic analysis for the absolute quantitation of the three proteins can provide greater confidence in the biomarkers. Exosomes have been studied in diagnosis and disease progression using proteomics in PD. 

## 5. Clinical Trials Using Proteomics for Biomarker Discovery in ALS and PD

The application of proteomics to patient stratification, biomarker measurements for clinical end points, and integration with genomics for the identification of novel drug targets has immense potential to advance precision medicine. Proteomic biomarkers have been implemented as a noninvasive diagnostic tool in many studies [117] and used patient stratification [117]. 

Developing a validated proteomic assay is critical prior to its utilization for decision making in the clinic. Discovery proteomics can identify thousands of proteins. However, to be able to validate these identified analytes, a small number is chosen, and a targeted proteomics strategy by MRM is often applied for absolute quantitation. Sometimes, protein signature classifiers identify groups of proteins, which can be used as biomarkers for patient stratification [118]. The Clinical Proteomics Tumor Analysis Consortium (CPTAC) has developed a fit-for-purpose best-practices guideline in targeted proteomic analysis for the validation of proteomic clinical assays [119]. These guidelines can be used to standardize targeted measurements for clinical use across diseases. The authors identified three tiers of analysis and described the validation steps for each tier with respect to the analytical goal of the assay. In Tier 1, the goal of the assay is to provide decision-making information in drug development or for medical practitioners on a small number of analytes. Since the intent is to use the assay for clinical purposes, a high degree of analytical validation is warranted, including measurements of assay precision, accuracy, specificity, analytical sensitivity (including limit of detection (LOD), limit of the blank (LOB), and lower limit of quantification (LLOQ)), linearity, and parallelism. The use of stable isotope internal standards for each analyte and/or protein heavy-labeled standards is recommended to achieve accurate quantitation. The other two tiers are for non-clinical purposes. Tier 2 uses stable isotopes to validate hundreds of analytes for research purposes. Tier 3, which is semi-quantitative, is used for exploratory studies [119]. Some examples of clinical studies that have used proteomics in ALS and PD are shown in Table 4.

## 6. Discussion

In ALS and PD, molecular biomarkers for diagnosis and prognosis are limited, and many of the available ones seem to be useful for advanced stages of the diseases, when therapeutic intervention is likely no longer effective. Both diseases have a critical need for biomarkers, and proteomics may be able to address this need. 

Current technological advancements in proteomics have made biomarker discovery for ALS and PD more efficient. Short gradients with novel LC systems [120] provide accurate and rapid quantitation without a loss of identifications with the new RTS-MS3 quantitation [121] or BoxCar DIA [31] to gain depth, range, and completeness, while addressing some of the dynamic range challenges in biofluids makes mass spectrometry a powerful and indispensable tool in biomarker discovery.

Mass spectrometry has advantages over immunoassays and aptamers because of its unbiased nature and non-reliance on antibody/aptamer specificity. This is particularly helpful in PTM analysis, in the identification of novel biomarkers, and in cases with point mutations or proteins, for which antibodies/aptamers are not available. In ALS, several PTMs have been defined in TDP43. These include hyperphosphorylation, polyubiquitination, and C-terminal truncations which are believed to play a role in aggregation and disease pathology [70]. In PD, α-synuclein is one of the best-studied proteins with several PTMs, including phosphorylation, nitration, ubiquitination, O-GlcNAcylation, and N- and C-terminal truncations. The use of mass shifts to characterize these PTMs and quantify their levels with mass spectrometry allows multiple PTMs to be analyzed in parallel in an unbiased manner without the need to synthesize and characterize antibodies/reagents. The characterization of disease-specific modifications in tissue and biofluids may serve as diagnostic markers or novel drug targets.

After comparing proteomic biomarker studies in tissue, biofluids, and exosomes in ALS, it was observed that proteins belonging to transcriptional pathways were altered in both spinal cord tissue and the CSF proteome. UCHL1, MAP2, and GPNMB were upregulated in spinal cord tissue and CSF in ALS [52]. Gelsolin was altered in ALS in plasma, and CSF was upregulated in CSF exosomes in patients with C9orf72 mutations. Clusterin was upregulated in CSF exosomes similar to CSF biofluids [56,61]. TDP43 modifications have been observed in human prefrontal, motor cortex brain tissue, and spinal cord, as well as in plasma-derived exosomes [51,55,63]. In PD, the presence of pS129 in α-synuclein is associated with the oligomeric form in brain tissues and has been observed in plasma, CSF and serum-derived neuronal exosomes (L1CAM+) in independent studies [90,101,103,116]. The total α-synuclein levels were upregulated in tissue and plasma-derived neuronal exosomes, but were unchanged in plasma measurements. The enrichment of exosomes helps to alleviate the dynamic range issue in proteomics of plasma/serum and could explain some findings where total α-synuclein levels in plasma were not significantly altered in PD, while neuronal-derived exosomes in plasma show upregulation in PD. Some studies comparing CSF and plasma in ALS demonstrated that gelsolin increased in both CSF and plasma. In PD, total and pS129 α-synuclein levels were measured in plasma and CSF [91]. An alternative is to use neuronal-derived exosomes from plasma for biomarker discovery. Further exploration and validation of neuronal specific markers in exosomes is warranted. 

While several of these biomarkers have been identified in certain patient populations such as ALS with C9orf72 mutations, the specificity of these biomarkers needs to be investigated to see if these markers will be useful in sporadic ALS or ALS with other mutations. To assess if C9orf72 biomarkers will be useful in a larger population, biomarker discovery and validation will need to be performed on non-C9orf72 patients. In a similar vein, the use of matched samples from siblings with mutations who do not exhibit symptoms can provide insight into prognostic markers.

When evaluating the value of biomarkers studies, animal models that are assessed longitudinally as a measure of disease progression might be useful. Similarly, identifying markers in patient cohorts could be validated in animal models or reprogrammed iPSC cells followed longitudinally. However, the caveat with using animal models particularly in neurodegenerative diseases is the low translatability. Several models do not accurately represent the disease pathology and systemic effects observed in patients. 

The other approach used to identify biomarkers that predict the onset of neurodegenerative diseases in the pre-symptomatic phase involves profiling healthy samples from people at risk of neurodegenerative diseases using mass spectrometry and by comparing signatures that change with the onset of symptoms and if these markers correlate with disease progression. The challenge lies in validating these early-onset biomarkers. Correlating molecular signatures to imaging markers is another avenue to identify early-onset markers. With growing technology and machine learning, biomarker signatures in the pre-symptomatic phase could potentially predict disease onset in the future. Efforts in the identification and validation of these markers in disease models with high translatability, as well as the assessment of early-onset samples, will remain a challenge in biomarker discovery for neurodegenerative diseases.

Proteomics has immense clinical potential for biomarker discovery, but there is only a limited number of validated proteomic biomarkers. This is due to the complexity and lack of standardized validation protocols for assays. In the cancer field, the use of validated targeted proteomic assays has been described by the CPTAC consortium. Applying these guidelines to biomarker validation in neurodegenerative diseases may be helpful in bridging the gap between discovery proteomics and the translation of biomarkers to the clinic. There are a few clinical trials that incorporate proteomics in their outcome measures in ALS and PD. Multi-omics studies integrating genomics, proteomics, transcriptomics, and metabolomics to identify markers for patient stratification may be able to address current challenges with biomarkers in diseases such as ALS and PD. 

## Figures and Tables

**Table 1:** Summary of methods used in proteomics.

Method	Advantages	Disadvantages
**Sample Preparation Methods**		
Filter-aided sample preparation (FASP)	Unbiased filter-based approach which removes detergents	Molecular weight cutoffs and can be challenging for aggregated proteins
Single-pot solid-phase-enhanced sample preparation (SP3)	Bead-based approach with low sample loss Unbiased robust recovery and can be automated using magnetic beads	Beads have a limited capacity and should not be overloaded to cause inconsistencies
In-StageTip (iST)	Peptide can be fractionated on the tip to gain depth	Tips must be compatible with solubilization reagents used
SarkoSpin	Can isolate insoluble pathological protein aggregated	Utilize detergents that require sample clean up prior to mass spectrometry
Depletion	Gain depth	Low throughput, induce variability
Immunoenrichment	Gain depth	Low throughput, induce variability
Offline fractionation	Gain depth	Low throughput
**Mass Spectrometry Quantitation Strategies**		
Isobaric TMT tags	Relative quantitation in MS2/MS3 dimension with multiplexing to save time	Require (SPS) MS3 for accurate quantitation to overcome challenges with ratio compression
Label-free DIA	Relative quantitation by area under the peak, enabling the acquisition of complete data in large cohorts	Requires expertise in MS method design for window strategy and data interpretation
MRM/PRM-targeted quantitation	Absolute quantitation with standard curves and the ability to monitor disease progression with time	Limited in the number of targets that can be analyzed
**Advances in Instrumentation and Technology**		
BOXCAR data-independent acquisition	Improves data completeness	Requires good method design
Real-time search RTS-SPS-MS3	Improves quantitation	Special feature in an instrument
High-field asymmetric ion mobility spectrometry FAIMS	Improves sensitivity and selectivity	In front end and not true ion mobility to separate isomers
Modified LC- Evosep	Improves throughput and robustness	Has defined methods and is not customizable
Automation	Improves reproducibility	Tedious to implement changes in workflows
Trapped ion mobility TIMS	PTM and isoform identification	Needs expertise to achieve good separation
Parallel acquisition serial fragmentation PASEF	Improves scan speed and sensitivity	Needs optimization based on gradient
Multiplexed immunoassay O-link	Improves dynamic range based on antibody specificity	Limited in the panel of targets based on the availability of antibodies
Aptamer-based assay	Improves dynamic range based on aptamer specificity	Limited in the panel of targets based on the availability of aptamers

**Table 2:** Proteomic studies in ALS focused on biomarker discovery.

Disease	Marker	Quantitation	Tissue	Summary	Reference
**Tissue-based proteomic markers in ALS**					
ALS	TDP43	PRM absolute quantitation	Prefrontal/motor cortex and spinal cord	An increase in C: N-terminal TDP43 peptide ratio > 1.5, new truncation site-specific trend observed in ALS-TDP	[51,55]
ALS (sporadic)	Calmodulin	Label-free	Spinal cord	Downregulated in ALS	[51]
ALS (sporadic)	ATP5D	Label-free	Spinal cord	Downregulated in ALS	[51]
ALS	UCHL1	Label-free and MRM	Spinal cord	Upregulated in ALS and correlated with CSF	[52]
ALS	MAP2	Label-free and MRM	Spinal cord	Upregulated in ALS and correlated with CSF	[52]
ALS	GPNMB	Label-free and MRM	Spinal cord	Upregulated in ALS and correlated with CSF	[52]
**Plasma/Serum proteomics biomarkers in ALS**					
ALS	Gelsolin	LFQ and MRM	Plasma	Differentially expressed in ALS	[56,57,58]
ALS	Clusterin	MRM	Plasma	Downregulated in ALS	[57,58]
ALS	CD5L	MRM	Plasma	Differentially expressed in ALS	[57,58]
ALS	Ficolin 3	MRM	Plasma	Upregulated in ALS	[57,58]
**CSF proteomic biomarkers in ALS**					
ALS	α-1-antichymotrypsin	LFQ	CSF	In CSF, 118 proteins were significantly altered in ALS compared to controls	[56]
ALS	Amyloid beta A4 protein	LFQ	CSF	In CSF, 118 proteins were significantly altered in ALS compared to controls	[56]
ALS	Gelsolin	LFQ	CSF	In CSF, 118 proteins were significantly altered in ALS compared to controls	[56]
ALS	Chitinase-3-like protein 1 (CHI3L1)	LFQ	CSF	Upregulated	[59]
ALS	Chitinase-3-like protein 2 (CHI3L2)	LFQ, TMT	CSF	Upregulated in mutated C9orf72 symptomatic ALS compared to asymptomatic controls with C9orf72 mutations	[59,60]
ALS	Chitotriosidase-1 (CHIT-1)	LFQ, TMT	CSF	Upregulated in mutated C9orf72 symptomatic ALS compared to asymptomatic controls with C9orf72 mutations	[59,60]
ALS	Ubiquitin carboxyl-terminal hydrolase isozyme L1 (UCHL1)	LFQ, TMT, MRM	CSF	Upregulated in mutated C9orf72 symptomatic ALS compared to asymptomatic controls with C9orf72 mutations	[52,59,60]
ALS	MAP2	MRM	CSF	Upregulated	[52]
ALS	CAPG	MRM	CSF	Upregulated	[52]
ALS	GPNMB	MRM	CSF	Upregulated	[52]
ALS	CRYAB	TMT	CSF	Upregulated in mutated C9orf72 symptomatic ALS compared to asymptomatic controls with C9orf72 mutations	[60]
ALS	PFN1	TMT	CSF	Upregulated in mutated C9orf72 symptomatic ALS compared to asymptomatic controls with C9orf72 mutations	[60]
ALS	TFRC	TMT	CSF	Upregulated in mutated C9orf72 symptomatic ALS compared to asymptomatic controls with C9orf72 mutations	[60]
ALS	TREM2	TMT	CSF	Upregulated in C9orf72 variant-associated symptomatic ALS compared to asymptomatic controls with C9orf72 variants	[60]
ALS	TXNDC17	TMT	CSF	Upregulated in mutated C9orf72 variant-associated symptomatic ALS compared to asymptomatic controls with C9orf72 variants	[60]
ALS	NEFM	TMT	CSF	Upregulated in mutated C9orf72 symptomatic ALS compared to asymptomatic controls with C9orf72 mutations	[60]
**Exosomal biomarkers in ALS**					
ALS	Gelsolin	LFQ	CSF exosomes	Upregulated in C9orf mutated ALS cases	[61]
ALS	Clusterin	LFQ	CSF exosomes	Upregulated	[61]
ALS	UBA1	LFQ	CSF exosomes	Upregulated in C9orf mutated ALS cases	[61]
ALS	NIR	LFQ	CSF exosomes	Upregulated in sporadic ALS	[62]
ALS	TDP43	LFQ	Plasma exosomes	Levels correlated with longitudinal progression	[63]

**Table 3:** Proteomic studies in PD focused on biomarker discovery.

Disease	Marker	Quantitation	Tissue	Summary	Reference
**Tissue-based proteomic markers in PD**					
PD	Mitochondrial dysfunction, oxidative stress, cytoskeleton impairment-related proteins	TMT	Substantia nigra	Significant changes in expression levels of 204 nigral proteins in human PD samples	[85]
PD	RGS6	LFQ	Substantia nigra (Lewy body pathology)	Changes in proteins related to (1) Arp2/3 complex-mediated actin nucleation; (2) synaptic function; (3) poly(A) RNA binding; (4) basement membrane and endothelium; and (5) hydrogen peroxide metabolic processes	[86]
PD	GANAB	LFQ	Substantia nigra (Lewy body pathology)	Changes in proteins related to (1) Arp2/3 complex-mediated actin nucleation; (2) synaptic function; (3) poly(A) RNA binding; (4) basement membrane and endothelium; and (5) hydrogen peroxide metabolic processes	[86]
PD	CD59	LFQ	Substantia nigra (Lewy body pathology)	Changes in proteins related to (1) Arp2/3 complex-mediated actin nucleation; (2) synaptic function; (3) poly(A) RNA binding; (4) basement membrane and endothelium; and (5) hydrogen peroxide metabolic processes	[86]
**Plasma/serum proteomic biomarkers in PD**					
PD	Apolipoprotein A1	iTRAQ	Plasma/serum	Downregulated in PD	[87,88]
PD	Apolipoprotein A-IV	LFQ	Plasma/serum	Downregulated in PD	[87,88]
PD	Apolipoprotein B	LFQ	Plasma	Downregulated in PD	[89]
PD	Apolipoprotein CI	LFQ	Plasma	Downregulated in PD	[89]
PD	Apolipoprotein CIII	LFQ	Plasma	Downregulated in PD	[89]
PD	Apolipoprotein C4	LFQ	Plasma	Downregulated in PD	[89]
PD	Apolipoprotein C4	LFQ	Plasma	Downregulated in PD	[89]
PD	Apolipoprotein M	LFQ	Plasma	Downregulated in PD	[89]
PD	Inter-alpha-trypsin inhibitor heavy	LFQ	Plasma/serum	Downregulated in PD	[87]
PD	Complement C4A	LFQ	Plasma/serum	Downregulated in PD	[87]
PD	Complement C4B	iTRAQ	Plasma/serum	Downregulated in PD	[87,88]
PD	Complement C3	LFQ	Plasma/serum	Downregulated in PD	[87]
PD	Haptoglobin	LFQ	Plasma	Downregulated in PD	[89]
PD	Clusterin	LFQ	Plasma/serum	Upregulated in PD	[87]
PD	Transthyretin	LFQ	Plasma/serum	Upregulated in PD	[87]
PD	Zinc α-2 glycoprotein	LFQ	Plasma/serum	Upregulated in PD	[87]
PD	Vitamin D binding protein	LFQ	Plasma/serum	Upregulated in PD	[87]
PD	Afamin	LFQ	Plasma/serum	Upregulated in PD	[87]
**CSF proteomic biomarkers in PD**					
PD	α-synuclein peptide (81–96)	MRM	CSF	α-Synuclein peptide altered in PD	[90]
PD	α-synuclein pS129	MRM	CSF	α-Synuclein pS129 correlates with disease severity	[91]
PD	Granins	DIA	CSF	Granins are downregulated in PD	[92]
**Exosomal biomarkers in PD**					
PD	α-synuclein	LFQ	Serum neuronal(L1CAM+) exosomes	Upregulated in prodromal and clinical PD compared to controls and other neurodegenerative diseases	[93]
PD	Clusterin	LFQ	Serum neuronal (L1CAM+) exosomes, plasma exosomes	Upregulated in FTD but not PD, served as a combined marker with α-synuclein and is downregulated in PD in plasma exosomes	[93,94]
PD	α-synuclein	SRM	Plasma neuronal (L1CAM+) exosomes	α-synuclein is upregulated in PD	[95,96]
PD	Complement C1r	LFQ	Plasma exosomes	Downregulated in PD	[94]
PD	Apolipoprotein A1	LFQ	Plasma exosomes	Downregulated in PD	[94]

**Table 4:** Examples of clinical trials using proteomics in ALS and PD.

Disease	Clinical Trial	Summary	Reference
ALS	NCT01948102	An observational study for the identification of prognostic and diagnostic markers in skin and adipose samples using proteomics to measure changes in abundance and/or post-translational modifications of proteins in the trial	[6]
PD	NCT00315250	An interventional study with the aim of developing imaging, clinical, and biochemical biomarkers for PD uses proteomics in combination with metabolomics and gene expression to categorize Parkinson’s syndrome vs. non-Parkinson’s syndrome	[7]
PD	NCT02263235	A study in Alzheimer’s, PD, and other neurological disorders without cognitive decline uses targeted quantitative proteomics by MRM in CSF, blood, urine, and saliva for diagnostic purposes after administering stable isotope-labelled leucine for the diagnosis of neurological disorders	[4]
PD	NCT02524405	An investigational study in Alzheimer’s and PD (called the brain–eye amyloid memory study (BEAM)), MRI, and amyloid PET were used for primary and secondary outcomes, genetic analysis for ApoE4 status, and proteomics and lipidomics analyses	[2]
PD	NCT02387281	An observational study in PD studying freezing of gait (FOG) proteomics on CSF is used in combination with analysis of catecholamines along with MRI and other cognitive tests to assess types of FOG and if there is a connection with cognitive differences and gait patterns presented in PD	[3]
